# Automated red blood cell exchange as an adjunctive treatment for severe* Plasmodium falciparum* malaria at the Vienna General Hospital in Austria: a retrospective cohort study

**DOI:** 10.1186/1475-2875-11-158

**Published:** 2012-05-07

**Authors:** Lorenz Auer-Hackenberg, Thomas Staudinger, Andja Bojic, Gottfried Locker, Gerda C Leitner, Wolfgang Graninger, Stefan Winkler, Michael Ramharter, Nina Worel

**Affiliations:** 1 Department of Medicine I, Division of Infectious Diseases and Tropical Medicine, Medical University of Vienna, Währinger Gürtel 18-20, 1190, Vienna, Austria; 2 Department of Medicine I, Intensive Care Unit, Medical University of Vienna, Vienna, Austria; 3 Department of Bloodgroup Serology and Transfusion Medicine, Medical University of Vienna, Vienna, Austria; 4 Institute for Tropical Medicine, University of Tübingen, Tübingen, Germany

**Keywords:** Malaria, Severe malaria, Plasmodium falciparum, Erythrocyte transfusion, Cytapheresis, Erythrocytapheresis, Automated red blood cell exchange, Whole blood exchange transfusion

## Abstract

**Background:**

Severe falciparum malaria is associated with considerable rates of mortality, despite the administration of appropriate anti-malarial treatment. Since overall survival is associated with total parasite biomass, blood exchange transfusion has been proposed as a potential method to rapidly reduce peripheral parasitaemia. However, current evidence suggests that this treatment modality may not improve outcome. Automated red blood cell exchange (also referred to as “erythrocytapheresis”) has been advocated as an alternative method to rapidly remove parasites from circulating blood without affecting patients’ volume and electrolyte status. However, only limited evidence from case reports and case series is available for this adjunctive treatment. This retrospective cohort study describes the use of automated red blood cell exchange for the treatment of severe malaria at the Medical University of Vienna.

**Methods:**

Epidemiologic data for imported malaria cases in Austria are reported and data of patients treated for malaria at the General Hospital/Medical University of Vienna were extracted from electronic hospital records.

**Results:**

Between 2000 and 2010, 146 patients were hospitalized at the Medical University of Vienna due to malaria and 16 of those were classified as severe malaria cases. Eleven patients of this cohort were potentially eligible for an adjunctive treatment with automated red blood cell exchange. Five patients eventually underwent this procedure within a period of seven hours (range: 3–19 hours) after hospital admission. Six patients did not undergo this adjunctive treatment following the decision of the treating physician. The procedure was well tolerated in all cases and rapid reduction in parasite counts was achieved without occurrence of haemodynamic complications. One patient died within seven days, whereas four patients survived without any sequelae.

**Discussion and conclusion:**

Automated red blood cell exchange was a safe and efficient procedure to rapidly clear peripheral parasitaemia. Whether the fast reduction in parasite biomass may ultimately improve patient survival remains however unclear. Randomized controlled trials are needed to conclusively appreciate the value of this adjunctive treatment.

## Background

Severe malaria, caused by *Plasmodium falciparum,* is associated with high mortality despite appropriate management at intensive care facilities. Few therapeutic interventions have been shown effective to improve survival, including parenteral artesunate, mechanical ventilation and renal replacement therapy [1-6]. Other adjunctive treatments (e.g. application of corticosteroids to reduce cerebral oedema, anticonvulsive drugs in cerebral malaria or antipyretics.) did not improve patient’s outcome, some of which were even associated with increased rates of sequelae and death [7-9].

Whole blood exchange transfusion has been proposed as a method to rapidly reduce parasite biomass [10]. A meta-analysis of eight case–control studies including 279 patients demonstrated that whole blood exchange transfusion was not superior to parenteral anti-malarial chemotherapy alone, discouraging the use of this adjunctive treatment [11].

Automated red blood cell (RBC) exchange is a technique that may potentially overcome problems associated with whole blood exchange [12-16]. With this procedure, RBCs are separated automatically from the patient’s whole blood, while plasma, platelets and white blood cells are retained. This procedure is known to be better tolerated in various indications compared to whole blood exchange transfusion with regards to volume alterations, coagulation and electrolyte disturbances, and susceptibility to infection [17-19]. RBC exchange has been successfully used in patients with sickle cell crises [20] and has been advocated as adjunctive treatment option for severe malaria. First case reports and case series have supported the use of automated RBC exchange and several national guidelines acknowledge the therapeutic potential for selected patients [21-25]. However, firm evidence for RBC exchange derived from randomized controlled clinical trials is still lacking. In the absence of such information, more data from case series and patient cohorts at different institutions are needed to increase the level of evidence. This study reports the use of RBC exchange as an adjunctive treatment for severe falciparum malaria in a cohort of patients treated at the Medical University of Vienna in Austria between 2000 and 2010.

## Methods

### Patient selection

Epidemiologic data on the number of malaria cases in Austria were obtained from mandatory reports of notifiable infectious diseases. According to Austrian law, all suspected, confirmed or fatal cases of malaria have to be reported to public authorities and the federal ministry of health, respectively. A list of all patients treated for malaria at the Medical University of Vienna was obtained from an automated database search of the institution’s electronic patient record system. Individual patient data were extracted from electronic hospital records. All clinical and laboratory data associated with the automated RBC exchange were obtained from patient record forms at the Department of Bloodgroup Serology and Transfusion Medicine. Records were entered into an electronic database. The study was approved by the Ethics Committee of the Medical University of Vienna. The World Health Organization (WHO) criteria for severe malaria [25] were employed for classification of severity of malaria cases. Patients received anti-malarial chemotherapy according to standard treatment guidelines based on randomized controlled trials, irrespective of the commencement of RBC exchange, with parenteral quinine and clindamycin [1,6,24,26].

### Automated RBC exchange

Automated RBC exchange was conducted by dedicated personnel of the Department of Bloodgroup Serology and Transfusion Medicine as an adjunctive treatment to anti-malarial chemotherapy. According to institutional consensus guidelines, patients diagnosed with acute *P. falciparum* malaria demonstrating hyperparasitaemia defined as >10 % infected erythrocytes and single or multi-organ failure were eligible for RBC exchange in addition to routine anti-malarial drug administration. For further classification, subsequent assessment of treatment responses and parasitaemia, Giemsa-stained thick and thin blood smears were prepared using standard protocols at admission, before and after RBC exchange, and regularly until specimen were free of parasites.

## Results

### Patient characteristics of malaria cases in Austria between 2000 and 2010

**Figure 1:**
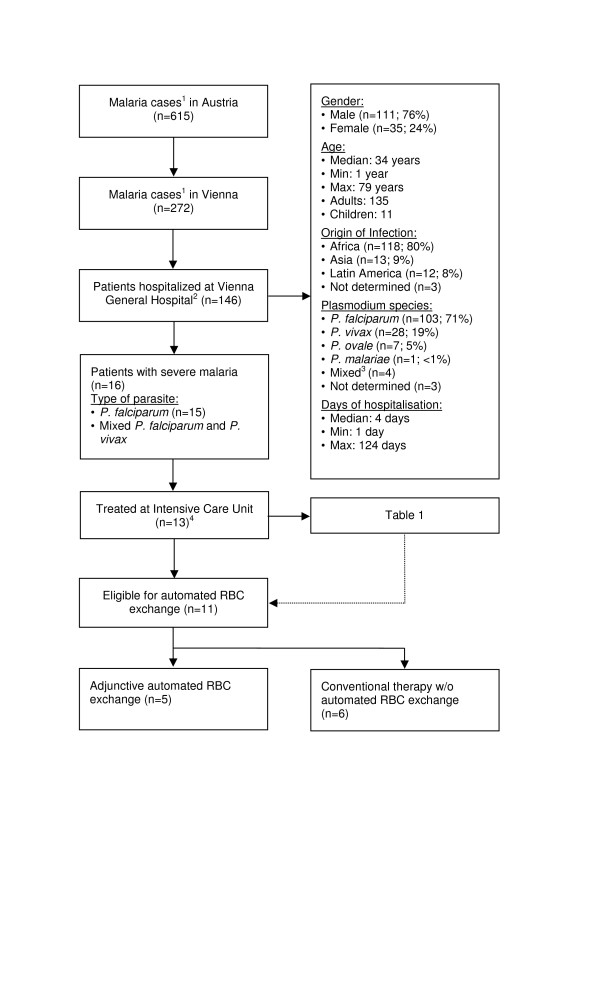
**Flow diagram of malaria cases in Austria and the Medical University of Vienna.**^1^ Reported to federal agencies; ^2^ According to the electronic patient record system; ^3^*Plasmodium falciparum* and *Plasmodium ovale* (n = 2), *Plasmodium falciparum* and *Plasmodium vivax* (n = 2); ^4^ Three patients did not require ICU admission (see text); ICU: intensive care unit; RBC: red blood cell.

Six hundred fifteen cases of malaria were reported to federal agencies in Austria between January 2000 and December 2010. Almost half of them (44 %, n = 272) occurred in the capital Vienna [27]. One hundred forty six patients (135 adults and 11 children) received in-patient treatment for malaria at the Medical University of Vienna during that time. Demographic data and patient characteristics are summarized in Figure 1. The majority (n = 111; 76 %) of patients were male and Africa was the most common place of infection (Nigeria n = 30; 20.5 %, Ghana n = 19; 13 % and Kenya n = 13; 8.9 %). Peripheral thick or thin blood smears showed acute *P. falciparum* infection in 107 cases (73.3 %), four of which were mixed infections with *Plasmodium vivax* (n = 2) or *Plasmodium ovale* (n = 2). Sixteen (11 %) patients suffered from severe malaria according to WHO criteria [1]. Thirteen (8.9 %) of those patients (Table 1) were treated at intensive care units (ICU) whereas the remaining three patients with severe malaria showed rapid improvement within less than 12 hours after initiation of therapy and were not transferred to an ICU.

**Table 1:** **Characteristics of patients with severe*Plasmodium falciparum*treated at the ICU with conventional treatment alone or with adjunctive automated RBC exchange**

		**Conventional treatment (n = 8)**	**Adjunct RBC exchange (n = 5)**
**Median Age in years (range)**		43 (1–64)	54 (30–71)
**Sex (Male / female)**		7 / 1	4 / 1
**Median days on ICU (range)**		6.5 (2–45)	7 (2–81)
**Ethnicity**	**Caucasian**	5	4
	**African**	2	-
	**Middle East**	1	1
**Area of Infection (n)**	**Africa**	7	5
	**Other**	1 (Nicaragua)	-
**Prophylaxis (n)**	**Yes**	-	-
	**No**	5	4
	**Not determined**	3	1
**Cerebral malaria (n)**		6 (75 %)	3 (60 %)
**Parasitaemia >10 % (n)**		6 (75 %)	5 (100 %)
**Mechanical ventilation (n)**		5 (63 %)	2 (40 %)
**Acute renal failure**^**1**^**(n)**		4 (50 %)	2 (40 %)
**Norepinephrine demand (n)**		4 (50 %)	4 (80 %)
**Baseline laboratory parameters (median, range)**	**Haemoglobin (g/dl)**	11.45 (4.9-16.1)	8.3 (5.7-12.6)
	**Platelets (G/l)**	28.5 (14–50)	46 (15–82)
	**Lactate (mmol/l)**	3.1 (2.2-5.02)	5.1 (2.3-12.9)
	**LDH (U/l)**	779 (278–1392)	692.5 (340–1848)
	**Blood glucose (mg/dl)**	123 (73–226)	85 (82–267)
**Median APACHE II score (range)**		17 (10–29)	26 (16–32)
**Median SAPS II (range)**		45 (34–93)	61 (39–78)
**Treatment**		Quinine + clindamycin	Quinine + clindamycin^3^
**Total number of RBC exchanges (n)**		0	7
·**Patients with 1 RBC exchange**		0	3
·**Patients with 2 RBC exchanges**		0	2
**Adverse Events (moderate or sever intensity)**		4	4
**Adverse events related to RBC exchange**		-	-
**Unrelated adverse events**		ARDS (n = 2)	ARDS (n = 1)
		Septic shock (n = 1)	VAP (n = 1)
		Retinopathy (n = 1)	Fungal sepsis (n = 1)
		Increase of intracranial pressure (n = 1)	DIC (n = 1)
		Pneumonia (n = 2)	Death (n = 1)
		Delirium (n = 2)	
		DIC (n = 2)	

^**1**^ Requiring renal replacement therapy; ^**2**^ Calculated after ICU admission within 24 hours; only applicable to adults; ^**3**^ Two patients initially received intra-rectal artesunate prior to referral; APACHE: acute physiology and chronic health evaluation; ARDS: acute respiratory distress; DIC: disseminated intravascular coagulation; ICU: intensive care unit; LDH: lactate dehydrogenase; RBC: red blood cell; SAPS: simplified acute physiology score; VAP: ventilator associated pneumonia.

### Patients presenting with severe *Plasmodium falciparum* infection

Fifteen patients fulfilling criteria for severe malaria had *P. falciparum* mono-infection and one patient showed mixed infection of *P. falciparum* and *P. vivax*. Intravenous anti-malarial chemotherapy (quinine dihydrochloride and clindamycin; 10 mg/kg every eighth hour with a loading dose of 20 mg/kg and 5–10 mg/kg twice daily, respectively) was initiated immediately after diagnosis. Electrocardiography, continuous blood glucose measurements and frequent clinical assessments during infusion ensured adequate monitoring for quinine related side effects. Eleven patients qualified for adjunctive RBC exchange according to the institution’s guidelines. Eventually, five adult patients received automated RBC exchange (Table 1). Six patients, though eligible, received anti-malarial chemotherapy alone following the decision of the treating physician. Since this was not a randomized controlled trial, the two treatment groups were not directly comparable. Most importantly, patients treated with adjunctive RBC exchange showed more advanced disease as evidenced by a higher median APACHE II score than patients treated with anti-malarial chemotherapy alone (26 versus 17; Table 1).

Eight of 13 patients with severe *P. falciparum* infection who were admitted to the ICU and treated with anti-malarial chemotherapy alone remained in hospital for a median of 24 days (range, 9–70) (Table 1). All but one patient had recently visited malaria endemic regions in Africa, five had not taken adequate anti-malarial chemoprophylaxis and three were unable to provide information about previous chemoprophylaxis. During the course of treatment, four patients developed acute renal failure and underwent haemodialysis. Furthermore, four patients required intermittent norepinephrine administration to maintain adequate blood pressures. Acute but mild retinopathy and increased intracranial pressure were each observed in one individual. One patient experienced acute respiratory distress syndrome (ARDS) and progressed to septic shock during recovery phase. However, all eight patients survived without clinical sequelae.

### Patients treated with adjunctive automated RBC exchange

Characteristics and clinical features of patients undergoing automated red blood cell exchange are shown in Table 1. All patients had recently visited malaria endemic regions in Africa, four had not taken adequate anti-malarial chemoprophylaxis and one patient was unable to provide information. RBC exchange was initiated within a median of seven hours (range, 3–19 hours) after first contact with a healthcare professional at the Medical University of Vienna. All five patients received leukocyte depleted, fully cross-matched packed red blood cells. For RBC exchange, the COM.TEC® (Fresenius, Bad Homburg) cell separator was used in the first patient (in 2001), whereas the COBE® Spectra apheresis system (CaridianBCT, Lakewood) was used in the other cases. RBC exchange was tolerated well without haemodynamic alterations or other side effects. During the apheresis procedure, a median 10 (range, 8–14) units of packed RBCs were necessary to replace 3068 ml (range, 2417–4591 ml) of potentially infected RBCs. RBC exchanges led to a rapid decrease in parasite counts to marginal levels (undetectable or <2 % infected RBCs) in peripheral blood smears after the first apheresis cycle in three patients and after a second cycle in the remaining two, respectively. Three patients experienced rapid clinical improvement and had an uncomplicated recovery without any sequelae or persistent disability. In two of these patients initial norepinephrine support was required to maintain a mean arterial blood pressure >60 mmHg and in another patient acute renal failure necessitated the use of haemodialysis. One patient developed cerebral malaria with significant neurological deficit (Glasgow Coma Scale 13), but fully regained consciousness without signs of persistent neurologic damage. One patient died eight days after admission and RBC exchange (APACHE II score at admission: 32). In this case, the delay of medical consultation following a febrile illness in Kenya led to unarousable coma during the patient’s return flight. When arriving at the ICU the patient had developed fully constricted, non-reactive pinpoint pupils and massive cerebral oedema (Glasgow Coma Scale 3). Another patient treated with automated RBC exchange had a prolonged and complicated recovery phase with renal failure, ARDS, ventilation associated pneumonia and fungal sepsis. Gradually, haemodialysis and mechanical ventilation could be discontinued after kidney and respiratory functions improved. Despite generalized weakness due to long term ICU admission, the patient did not develop persistent neurologic impairment. After 81 days of intensive care and 127 days admission in hospital, this patient could be discharged and transferred to a rehabilitation facility.

## Discussion

This study describes a cohort of patients undergoing RBC exchange in a tertiary health care institution in Austria. Out of 146 patients treated for malaria at the Medical University of Vienna, 13 were treated at an ICU department. Eleven of them fulfilled institutional criteria to consider RBC exchange, but only five patients were actually treated with automated RBC exchange in a ten-year period. This constitutes less than half of the patients (45 %) who were potentially eligible according to institution guidelines [24]. This discrepancy is most likely explained by logistical constraints of automated RBC exchange rather than by medical contraindications. Challenges include rapid transfer of patients to specialized medical centres, quick and reliable blood smear assessment, diagnosis by experienced physicians and established collaborations between infectious disease departments, departments for Transfusion Medicine, and the ICU. Furthermore, due to the lack of high quality evidence for automated RBC exchange and the questionable benefit of whole blood exchange [11], some physicians may opt against the use of this adjunctive treatment in severe malaria or may consider it too late for any potential benefit. A well-organized interdisciplinary approach involving staff of the departments of infectious diseases, ICU, and Transfusion Medicine is needed to address these logistical difficulties in a timely manner and encourage physicians to consider automated RBC exchange as an adjunctive treatment option.

Automated RBC exchange was commenced within an acceptable time period and the procedure was well tolerated without any signs and symptoms of clinical deterioration, electrolyte disturbances or bleeding complications. The efficacy of physical removal of parasites was shown by rapid clearance of peripheral parasitaemia. Despite the exchange volume of approximately 1–1.5 times of patients total RBC pool, apheresis did not lead to problems in fluid overload and haemodynamic distress, a phenomenon which was more frequently observed in patients undergoing whole blood exchange transfusion [17-19]. Moreover, during or shortly after automated RBC exchange no specific medical intervention became necessary to maintain haemodynamic and respiratory stability. The occurrence of pneumonia and subsequent lung failure in the patient with prolonged recovery was related to mechanical ventilation. Based on the clinical characteristics and the time course a causal association of this complication with RBC exchange was deemed unlikely. Several weeks after RBC exchange and completion of anti-malarial treatment, this patient experienced fungal sepsis, which was again a complication of prolonged hospitalization at an ICU. One patient in the adjunctive treatment group presented with advanced cerebral malaria and died at the ICU.

In summary, one out of five patients treated with automated RBC exchange died whereas all eight patients in the control group survived in this retrospective cohort study (Table 1). However, no direct comparison of survival rates between groups is justified since the absence of randomization led to preferential inclusion of patients with more advanced diseases in the RBC exchange group than in the conventional treatment arm as evidenced by the APACHE II score and lactate levels (Table 1).

Automated RBC exchange has been shown to be a safe method to rapidly reduce parasitaemia in critical ill patients. Since high-quality evidence from randomized controlled trials is missing, a final judgement on the risk benefit ratio of this adjunctive treatment for severe malaria is not possible to date. Although total biomass of parasites has been shown to correlate with severity of falciparum malaria [28,29], it remains unclear whether physical removal of parasites can improve patient’s outcome. However, it may be argued that rapid reduction of parasitaemia may benefit most patient populations at highest risk for adverse outcome including non-immune travellers and people living in regions of unstable malaria transmission [11,30]. Importantly, this risk benefit balance may depend on the employed anti-malarial drug and its speed of action. Whereas artemisinins show rapid parasite reduction in most endemic areas, quinine and clindamycin have a significantly slower onset of activity [2,6,26,31,32]. However, parasite clearance times of artemisinin derivatives are dramatically prolonged in regions of South East Asia harbouring artemisinin resistant parasite strains, indicating again a potential shift in the risk benefit ratio of this potential adjunctive treatment in different regions of the world [33,34].

Limitations of this report include most importantly the retrospective study design since this does not guarantee comparability of patient groups in the absence of randomization and unified consistent definitions in the malaria patients’ general care (e.g indications for respiratory support, ventilation or renal replacement therapy may have slightly varied within ten years and between different intensive care facilities). Improving the level of evidence is therefore important; however the conduction of prospective studies evaluating this intervention is inherently difficult since high transmission regions often lack resources for purchasing and maintenance of equipment and the availability of safe blood products. Resource rich regions on the other hand generally do not have adequate numbers of patients with this condition. It is, therefore, likely that cohort studies will remain the main source of evidence in the near future.

## Conclusion and outlook

Whereas the reported experience with automated RBC exchange is comparable to previous case reports [12-17], the reporting of patient cohorts puts this adjunctive treatment in perspective by providing information about the proportion of patients undergoing this procedure at the Medical University of Vienna. Since randomized controlled trials are unlikely to be conducted in the near future, further cohort studies are the best way forward to increase the still limited knowledge and experience with this adjunctive treatment at this stage. This will allow to gradually increasing the level of evidence and will ultimately help to determine whether and which patient populations may benefit from this adjunctive treatment.

## Competing interests

The authors declare that they have no competing interests.

## Authors’ contribution

LAH, NW, SW and MR have designed the study, were responsible for data acquisition, performed data analysis and drafted the manuscript. All authors have reviewed and approved the final version of the manuscript.
